# Supplementary material for: A SENtence Supramodal Areas AtlaS (SENSAAS) based on multiple task-induced activation mapping and graph analysis of intrinsic connectivity in 144 healthy right-handers
Source: Brain Struct Funct. 2018 Dec 7;224(2):859–82. doi: 10.1007/s00429-018-1810-2 (PMC6420474; doi:10.1007/s00429-018-1810-2)
Supplement: Supplementary file 1 — Supplementary material 1 (DOCX 45 KB) [file 429_2018_1810_MOESM1_ESM.docx]

**Supplementary Table 1.** Comparison of 4 different clustering methods (agglomerative Hierarchical Cluster Analysis with Ward’s and average distance method, Gaussian mixture model and k-means) applied on the set of 32 hROIs co-activated and co-leftward asymmetrical during the sentence tasks. In aHCA, we chose the number of clusters that fulfilled a maximum of indices amongst the 30 statistical indices of the R library “NbClust”. The number of clusters in Gaussian mixture model and k-means was set according to the number of clusters find in aHCA. The last line: “aRi” (adjusted Rand index), corresponds to the classification comparison between the aHCA with Ward’s method and the 3 others. hROIs in bold are those composed SENT_CORE.

|  | AICHA hROI | aHCA  (Ward’s distance) | aHCA  (average distance) | Gaussian  mixture model | k-means |
| --- | --- | --- | --- | --- | --- |
| Frontal & insula | prec3 | SENT_MEM | SENT_MEM | SENT_VISU | SENT_VISU |
|  | **prec4** | **SENT_CORE** | **SENT_CORE** | **SENT_CORE** | **SENT_CORE** |
|  | **F1_2** | **SENT_CORE** | **SENT_CORE** | **SENT_CORE** | **SENT_CORE** |
|  | **f2_2** | **SENT_CORE** | **SENT_CORE** | **SENT_CORE** | **SENT_VISU** |
|  | **F3t** | **SENT_CORE** | **SENT_CORE** | **SENT_CORE** | **SENT_CORE** |
|  | **F3O1** | **SENT_CORE** | **SENT_CORE** | **SENT_CORE** | **SENT_CORE** |
|  | INSa1 | SENT_VISU | SENT_VISU | SENT_VISU | SENT_VISU |
|  | **INSa2** | **SENT_CORE** | **SENT_CORE** | **SENT_CORE** | **SENT_CORE** |
|  | **INSa3** | **SENT_CORE** | **SENT_CORE** | **SENT_CORE** | **SENT_VISU** |
| Temporal & parietal | **T1_4** | **SENT_CORE** | **SENT_CORE** | **SENT_CORE** | **SENT_VISU** |
|  | **T2_3** | **SENT_CORE** | **SENT_CORE** | **SENT_CORE** | **SENT_CORE** |
|  | **T2_4** | **SENT_CORE** | **SENT_CORE** | **SENT_CORE** | **SENT_VISU** |
|  | T3_4 | SENT_VISU | SENT_VISU | SENT_VISU | SENT_VISU |
|  | **STS1** | **SENT_CORE** | **SENT_CORE** | **SENT_CORE** | **SENT_CORE** |
|  | **STS2** | **SENT_CORE** | **SENT_CORE** | **SENT_CORE** | **SENT_CORE** |
|  | **STS3** | **SENT_CORE** | **SENT_CORE** | **SENT_CORE** | **SENT_CORE** |
|  | **STS4** | **SENT_CORE** | **SENT_CORE** | **SENT_CORE** | **SENT_CORE** |
|  | **SMG7** | **SENT_CORE** | **SENT_CORE** | **SENT_CORE** | **SENT_CORE** |
|  | **AG2** | **SENT_CORE** | **SENT_CORE** | **SENT_MEM** | **SENT_MEM** |
|  | O3_1 | SENT_VISU | SENT_VISU | SENT_VISU | SENT_VISU |
|  | FUS4 | SENT_VISU | SENT_VISU | SENT_VISU | SENT_VISU |
|  | pHIPP1 | SENT_VISU | SENT_VISU | SENT_VISU | SENT_MEM |
|  | HIPP2 | SENT_MEM | SENT_MEM | SENT_MEM | SENT_MEM |
| Internal surface | **SMA2** | **SENT_CORE** | **SENT_CORE** | **SENT_CORE** | **SENT_CORE** |
|  | **SMA3** | **SENT_CORE** | **SENT_CORE** | **SENT_CORE** | **SENT_CORE** |
|  | pCENT4 | SENT_MEM | SENT_MEM | SENT_MEM | SENT_MEM |
|  | CINGp3 | SENT_MEM | SENT_MEM | SENT_MEM | SENT_MEM |
|  | PRECU6 | SENT_MEM | SENT_MEM | SENT_MEM | SENT_MEM |
| Sub-cortical | AMYG | SENT_VISU | SENT_VISU | SENT_VISU | SENT_VISU |
|  | THA4 | SENT_MEM | SENT_MEM | SENT_MEM | SENT_MEM |
|  | PUT2 | SENT_VISU | SENT_VISU | SENT_VISU | SENT_VISU |
|  | PUT3 | SENT_VISU | SENT_VISU | SENT_CORE | SENT_VISU |
| aRi | - | - | 1 | 0.76 | 0.43 |

**Supplementary Table 2.** SENT_CORE hROIs correlations (R) between the degree centrality (DC) values calculated across the 185 hROIs of the left hemisphere and the mean activation in each of the 3 language tasks. hROIs with a star (*) are those having a significant correlation (p < 0.05) between the language tasks activation values and DC values.

|  | AICHA hROI | MANCOVA | | PROD_SENT-WORD_ | | LISN_SENT-WORD_ | | READ_SENT-WORD_ | |
| --- | --- | --- | --- | --- | --- | --- | --- | --- | --- |
|  |  | DC | DC*Task | R | p | R | p | R | p |
| Frontal & insula | prec4 | 0.13 | 0.10 | -0.190 | 0.026 | -0.107 | 0.21 | 0.012 | 0.89 |
|  | F1_2 | 0.060 | 0.060 | 0.168 | 0.049 | 0.008 | 0.93 | 0.153 | 0.073 |
|  | f2_2 | 0.67 | 0.11 | -0.096 | 0.26 | 0.084 | 0.33 | 0.110 | 0.20 |
|  | F3t* | 0.024 | 0.16 | 0.161 | 0.058 | 0.079 | 0.36 | 0.190 | 0.025 |
|  | F3O1 | 0.60 | 0.43 | -0.018 | 0.83 | 0.018 | 0.83 | -0.092 | 0.28 |
|  | INSa2 | 0.95 | 0.071 | -0.007 | 0.93 | -0.139 | 0.10 | 0.099 | 0.25 |
|  | INSa3 | 0.93 | 0.43 | -0.039 | 0.65 | -0.019 | 0.82 | 0.080 | 0.35 |
| Temporal & parietal | T1_4 | 0.52 | 0.24 | 0.031 | 0.72 | -0.150 | 0.078 | -0.036 | 0.68 |
|  | T2_3 | 0.19 | 0.21 | 0.143 | 0.095 | 0.008 | 0.93 | 0.096 | 0.26 |
|  | T2_4 | 0.47 | 0.83 | -0.079 | 0.35 | -0.061 | 0.48 | -0.025 | 0.77 |
|  | STS1 | 0.092 | 0.42 | 0.156 | 0.068 | 0.078 | 0.36 | 0.106 | 0.22 |
|  | STS2 | 0.74 | 0.32 | 0.066 | 0.44 | -0.038 | 0.66 | 0.040 | 0.64 |
|  | STS3 | 0.15 | 0.12 | 0.162 | 0.057 | 0.031 | 0.72 | 0.092 | 0.28 |
|  | STS4 | 0.24 | 0.79 | -0.096 | 0.26 | -0.121 | 0.16 | -0.056 | 0.52 |
|  | SMG7* | 0.050 | 0.42 | 0.161 | 0.058 | 0.110 | 0.20 | 0.138 | 0.11 |
|  | AG2 | 0.90 | 0.87 | 0.029 | 0.73 | -0.021 | 0.80 | 0.013 | 0.88 |
| Internal surface | SMA2 | 0.28 | 0.19 | 0.084 | 0.33 | -0.022 | 0.80 | 0.135 | 0.11 |
|  | SMA3 | 0.61 | 0.49 | -0.067 | 0.44 | -0.043 | 0.61 | 0.035 | 0.69 |
